# 5-(4-Bromo­anilinomethyl­ene)-2,2-dimethyl-1,3-dioxane-4,6-dione

**DOI:** 10.1107/S1600536809035776

**Published:** 2009-09-12

**Authors:** Jian-You Shi, Jin-Cheng Yang, Jin-Liang Yang

**Affiliations:** a Department of Medicinal Chemistry, West China School of Pharmacy, Sichuan University, Chengdu 610041, People’s Republic of China; b State Key Laboratory of Biotherapy, West China Hospital, Sichuan University, Chengdu 610041, People’s Republic of China

## Abstract

In the title compound, C_13_H_12_BrNO_4_, the dihedral angles between the amino­methyl­ene group and the dioxane ring and between the benzyl ring and the amino­methyl­ene unit are  7.96 (4) and 12.15 (4)°, respectively. The dioxane ring shows a half-boat conformation, in which the C atom between the dioxane ring O atoms is 0.460 (8) Å out of the plane through the remaining ring atoms. An intra­molecular N—H⋯O hydrogen bond may stabilize the planar conformation of the mol­ecule. An inter­molecular C—H⋯O inter­action is also present.

## Related literature

For the synthesis of related compounds, see: Cassis *et al.* (1985 ▶). For the synthesis of related anti­tumor precursors, see: Ruchelman *et al.* (2003 ▶). For the crystal structures of related 5–aryl­amino­methyl­ene–2,2–dimeth­yl–1,3–dioxane–4,6–dione derivatives, see: Li *et al.* (2009**a* ▶,*b* ▶,c* ▶). 
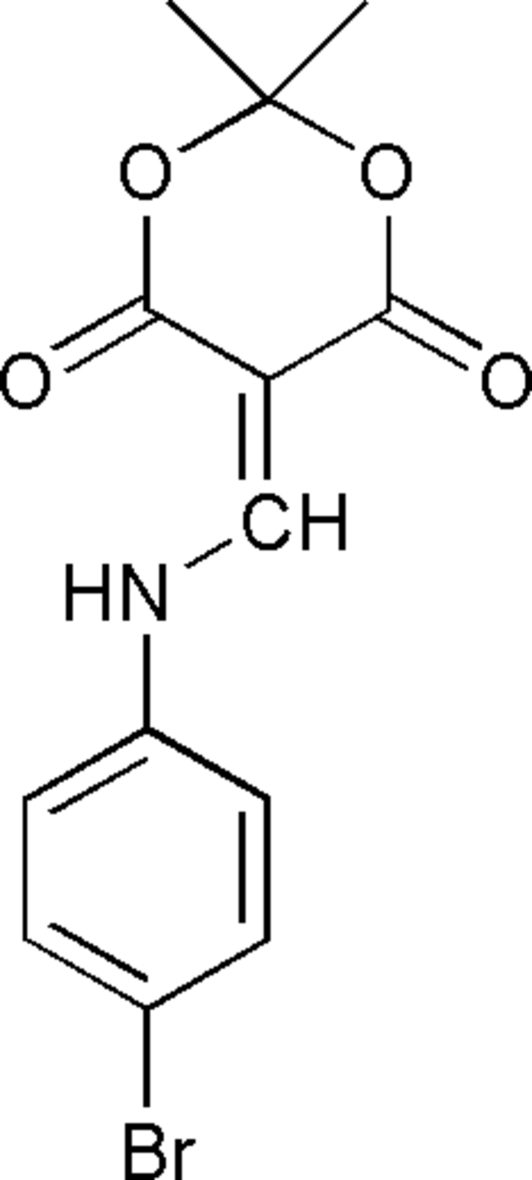


## Experimental

### 

#### Crystal data

C_13_H_12_BrNO_4_
*M* *_r_* = 326.14
Monoclinic, 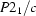

*a* = 13.837 (3) Å
*b* = 13.019 (3) Å
*c* = 7.4900 (15) Å
β = 105.24 (3)°
*V* = 1301.8 (5) Å^3^
*Z* = 4
Mo *K*α radiation
μ = 3.17 mm^−1^
*T* = 113 K
0.20 × 0.18 × 0.04 mm

#### Data collection

Rigaku Saturn CCD area-detector diffractometer
Absorption correction: multi-scan (*CrystalClear*; Rigaku/MSC, 2005 ▶) *T*_min_ = 0.570, *T*_max_ = 0.884
9108 measured reflections
2279 independent reflections
1063 reflections with *I* > 2σ(*I*)
*R*_int_ = 0.113

#### Refinement

*R*[*F* ^2^ > 2σ(*F* ^2^)] = 0.066
*wR*(*F* ^2^) = 0.160
*S* = 0.99
2279 reflections
179 parameters
H atoms treated by a mixture of independent and constrained refinement
Δρ_max_ = 0.97 e Å^−3^
Δρ_min_ = −0.96 e Å^−3^

### 

Data collection: *CrystalClear* (Rigaku/MSC, 2005 ▶); cell refinement: *CrystalClear*; data reduction: *CrystalClear*; program(s) used to solve structure: *SHELXS97* (Sheldrick, 2008 ▶); program(s) used to refine structure: *SHELXL97* (Sheldrick, 2008 ▶); molecular graphics: *ORTEP-3* (Farrugia, 1997 ▶); software used to prepare material for publication: *SHELXL97* and *PLATON* (Spek, 2009 ▶).

## Supplementary Material

Crystal structure: contains datablocks I, global. DOI: 10.1107/S1600536809035776/rk2160sup1.cif

Structure factors: contains datablocks I. DOI: 10.1107/S1600536809035776/rk2160Isup2.hkl

Additional supplementary materials:  crystallographic information; 3D view; checkCIF report

## Figures and Tables

**Table 1:** Hydrogen-bond geometry (Å, °)

*D*—H⋯*A*	*D*—H	H⋯*A*	*D*⋯*A*	*D*—H⋯*A*
N1—H1*N*⋯O4	0.98 (7)	2.06 (8)	2.770 (7)	128 (6)
C9—H9⋯O3^i^	0.93	2.49	3.345 (8)	152

Symmetry code: (i) 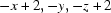.

**Fig. 1.**
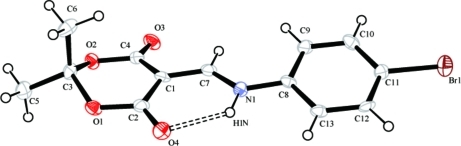
The molecular structure of the title compound with the numbering scheme. Displacement ellipsoids are drawn at the 30% probability level. H atoms are presented as a small spheres of arbitrary radius.

**Fig. 2.**
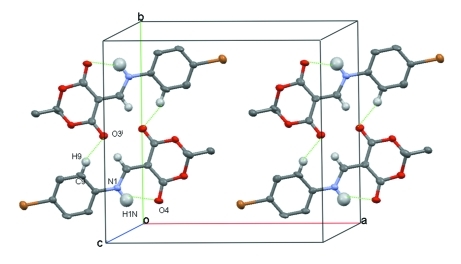
Crystal packing of the title compound, showing the intermolecular hydrogen bonds as dashed lines. H atoms not involved in these interactions have been omitted. Symmetry code: (i) -x+2, -y, -z+2.

## supplementary crystallographic information

### Comment

The 4(1*H*)quinolone structure plays an extremely important role in the field of pharmaceutical chemistry. The 5–arylaminomethylene–2,2–dimethyl–1,3–dioxane–4,6–diones are the key intermediates which can be used to synthesize the 4(1*H*)quinolone derivatives by thermolysis (Cassis *et al.*, 1985), that can be used as precursors foranti–malarial agents, anticancer agents, and reversible (H+/K+) ATPase inhibitors (Ruchelman *et al.*, 2003). The conformation of the title compound is similar with those reported early by Li *et al.*, (2009*a,b,c*), which is almost planar with the dihedral angles of 7.96 (4)° and 12.15 (4)° between the aminomethylene group and the dioxane ring, and between the benzyl ring and the aminomethylene unit, respectively. Besides, the dioxane ring of the title compound exhibits a half–boat conformation, in which the C atom between the dioxane oxygen atoms is -0.460 (8) Å out–of–plane. The intramolecular N—H···O hydrogen bond (Table 1) is stabilizing the planar conformation in the molecule.

### Experimental

A solution of 2,2–dimethyl–1,3–dioxane–4,6–dione (1.44 g, 0.01 mol) and methylorthoformate (1.27 g, 0.012 mol) was heated to reflux for 2.5 h, then the arylamine (1.32 g, 0.01 mol) was added into the above solution. The mixture was heated under reflux for another 4 h and then filtered. Single crystals were obtained from the filtrate after 2 days.

### Refinement

The imino H atom was located in a difference Fourier map and refined isotropically. Other H atoms were positioned geometrically with C—H = 0.93Å for aromatic or 0.96Å for methyl, and refined using a riding model with *U*_iso_(H) = 1.5*U*_eq_(C) for methyl and 1.2*U*_eq_(C) for the others.

### Crystal data

**Table:**

C_13_H_12_BrNO_4_	*F*(000) = 656
*M**_r_* = 326.14	*D*_x_ = 1.664 Mg m^−^^3^
Monoclinic, *P*2_1_/*c*	Mo *K*α radiation, λ = 0.71073 Å
Hall symbol: -P 2ybc	Cell parameters from 4079 reflections
*a* = 13.837 (3) Å	θ = 2.2–27.7°
*b* = 13.019 (3) Å	µ = 3.17 mm^−^^1^
*c* = 7.4900 (15) Å	*T* = 113 K
β = 105.24 (3)°	Plate, colourless
*V* = 1301.8 (5) Å^3^	0.20 × 0.18 × 0.04 mm
*Z* = 4	

### Data collection

**Table:**

Rigaku Saturn CCD area-detector diffractometer	2279 independent reflections
Radiation source: rotating anode	1063 reflections with *I* > 2σ(*I*)
confocal	*R*_int_ = 0.113
Detector resolution: 7.31 pixels mm^-1^	θ_max_ = 25.0°, θ_min_ = 2.2°
ω and φ scans	*h* = −16→16
Absorption correction: multi-scan (*CrystalClear*; Rigaku/MSC, 2005)	*k* = −15→15
*T*_min_ = 0.570, *T*_max_ = 0.884	*l* = −7→8
9108 measured reflections	

### Refinement

**Table:**

Refinement on *F*^2^	Secondary atom site location: difference Fourier map
Least-squares matrix: full	Hydrogen site location: mixed
*R*[*F*^2^ > 2σ(*F*^2^)] = 0.066	H atoms treated by a mixture of independent and constrained refinement
*wR*(*F*^2^) = 0.160	*w* = 1/[σ^2^(*F*_o_^2^) + (0.0585*P*)^2^] where *P* = (*F*_o_^2^ + 2*F*_c_^2^)/3
*S* = 0.99	(Δ/σ)_max_ = 0.001
2279 reflections	Δρ_max_ = 0.97 e Å^−^^3^
179 parameters	Δρ_min_ = −0.96 e Å^−^^3^
0 restraints	Extinction correction: *SHELXL97* (Sheldrick, 2008), Fc^*^=kFc[1+0.001xFc^2^λ^3^/sin(2θ)]^-1/4^
Primary atom site location: structure-invariant direct methods	Extinction coefficient: 0.017 (3)

### Special details

**Table:**

Geometry. All s.u.'s (except the s.u. in the dihedral angle between two l.s. planes) are estimated using the full covariance matrix. The cell s.u.'s are taken into account individually in the estimation of s.u.'s in distances, angles and torsion angles; correlations between s.u.'s in cell parameters are only used when they are defined by crystal symmetry. An approximate (isotropic) treatment of cell s.u.'s is used for estimating s.u.'s involving l.s. planes.
Refinement. Refinement of *F*^2^ against ALL reflections. The weighted *R*–factor *wR* and goodness of fit *S* are based on *F*^2^, conventional *R*–factors *R* are based on *F*, with *F* set to zero for negative *F*^2^. The threshold expression of *F*^2^ > σ(*F*^2^) is used only for calculating *R*–factors(gt) *etc*. and is not relevant to the choice of reflections for refinement. *R*–factors based on *F*^2^ are statistically about twice as large as those based on *F*, and *R*–factors based on ALL data will be even larger.

### Fractional atomic coordinates and isotropic or equivalent isotropic displacement parameters (Å^2^)

**Table:**

	*x*	*y*	*z*	*U*_iso_*/*U*_eq_	
Br1	0.55042 (5)	0.35263 (5)	1.07667 (10)	0.0385 (4)	
O1	1.2599 (3)	0.2261 (3)	0.9075 (6)	0.0281 (11)	
O2	1.2115 (3)	0.0519 (3)	0.8410 (5)	0.0263 (11)	
O3	1.0562 (3)	0.0009 (3)	0.8058 (6)	0.0335 (12)	
O4	1.1515 (3)	0.3439 (3)	0.9505 (6)	0.0310 (12)	
N1	0.9664 (4)	0.2768 (4)	0.9800 (7)	0.0235 (13)	
H1N	1.008 (7)	0.337 (5)	0.978 (11)	0.08 (3)*	
C1	1.0970 (5)	0.1708 (4)	0.9159 (8)	0.0232 (16)	
C2	1.1680 (5)	0.2526 (5)	0.9294 (8)	0.0269 (16)	
C3	1.2909 (5)	0.1209 (5)	0.9352 (9)	0.0270 (16)	
C4	1.1175 (5)	0.0689 (5)	0.8546 (8)	0.0260 (16)	
C5	1.3741 (5)	0.1084 (5)	0.8402 (9)	0.0324 (17)	
H5A	1.3500	0.1275	0.7124	0.049*	
H5B	1.3955	0.0380	0.8485	0.049*	
H5C	1.4294	0.1516	0.8994	0.049*	
C6	1.3222 (5)	0.0959 (5)	1.1391 (8)	0.0351 (18)	
H6A	1.3725	0.1438	1.2018	0.053*	
H6B	1.3490	0.0275	1.1562	0.053*	
H6C	1.2652	0.1004	1.1890	0.053*	
C7	1.0035 (5)	0.1867 (5)	0.9435 (8)	0.0250 (16)	
H7	0.9624	0.1294	0.9362	0.030*	
C8	0.8705 (5)	0.2914 (5)	1.0096 (7)	0.0212 (15)	
C9	0.8101 (5)	0.2096 (5)	1.0358 (8)	0.0294 (17)	
H9	0.8340	0.1426	1.0388	0.035*	
C10	0.7156 (5)	0.2274 (5)	1.0571 (8)	0.0284 (16)	
H10	0.6755	0.1730	1.0746	0.034*	
C11	0.6813 (5)	0.3276 (5)	1.0521 (8)	0.0241 (16)	
C12	0.7412 (5)	0.4097 (4)	1.0325 (8)	0.0259 (16)	
H12	0.7180	0.4766	1.0339	0.031*	
C13	0.8362 (5)	0.3912 (5)	1.0109 (8)	0.0252 (16)	
H13	0.8768	0.4459	0.9973	0.030*	

### Atomic displacement parameters (Å^2^)

**Table:**

	*U*^11^	*U*^22^	*U*^33^	*U*^12^	*U*^13^	*U*^23^
Br1	0.0334 (6)	0.0385 (6)	0.0460 (6)	0.0036 (3)	0.0148 (4)	−0.0078 (3)
O1	0.038 (3)	0.020 (2)	0.031 (3)	0.000 (2)	0.019 (2)	0.000 (2)
O2	0.030 (3)	0.022 (2)	0.032 (3)	0.001 (2)	0.016 (2)	−0.004 (2)
O3	0.042 (3)	0.022 (3)	0.044 (3)	−0.005 (2)	0.024 (3)	−0.001 (2)
O4	0.040 (3)	0.018 (3)	0.038 (3)	0.000 (2)	0.016 (2)	−0.006 (2)
N1	0.028 (4)	0.019 (3)	0.025 (3)	0.001 (3)	0.010 (3)	−0.003 (2)
C1	0.035 (5)	0.019 (4)	0.019 (3)	−0.003 (3)	0.013 (3)	0.004 (3)
C2	0.037 (5)	0.027 (4)	0.019 (3)	0.006 (3)	0.012 (3)	0.005 (3)
C3	0.029 (5)	0.020 (4)	0.034 (4)	0.008 (3)	0.012 (3)	−0.003 (3)
C4	0.030 (4)	0.023 (4)	0.027 (4)	0.003 (3)	0.010 (3)	0.006 (3)
C5	0.034 (5)	0.023 (4)	0.043 (4)	0.002 (3)	0.014 (4)	−0.006 (3)
C6	0.047 (5)	0.030 (4)	0.024 (4)	0.015 (3)	0.002 (3)	−0.004 (3)
C7	0.034 (5)	0.024 (4)	0.019 (3)	0.002 (3)	0.010 (3)	0.005 (3)
C8	0.030 (4)	0.020 (4)	0.014 (3)	−0.004 (3)	0.008 (3)	−0.002 (3)
C9	0.047 (5)	0.018 (4)	0.028 (4)	0.009 (3)	0.019 (3)	0.000 (3)
C10	0.037 (5)	0.030 (4)	0.022 (3)	−0.006 (3)	0.014 (3)	−0.005 (3)
C11	0.028 (4)	0.022 (4)	0.022 (4)	0.001 (3)	0.007 (3)	−0.003 (3)
C12	0.042 (5)	0.013 (3)	0.023 (4)	0.008 (3)	0.010 (3)	0.005 (3)
C13	0.041 (5)	0.016 (3)	0.022 (4)	−0.001 (3)	0.014 (3)	0.000 (3)

### Geometric parameters (Å, °)

**Table:**

Br1—C11	1.895 (6)	C5—H5B	0.9600
O1—C2	1.369 (7)	C5—H5C	0.9600
O1—C3	1.433 (7)	C6—H6A	0.9600
O2—C4	1.350 (7)	C6—H6B	0.9600
O2—C3	1.449 (7)	C6—H6C	0.9600
O3—C4	1.214 (7)	C7—H7	0.9300
O4—C2	1.229 (6)	C8—C13	1.385 (8)
N1—C7	1.338 (7)	C8—C9	1.399 (8)
N1—C8	1.415 (8)	C9—C10	1.378 (8)
N1—H1N	0.98 (7)	C9—H9	0.9300
C1—C7	1.378 (8)	C10—C11	1.386 (8)
C1—C2	1.434 (8)	C10—H10	0.9300
C1—C4	1.455 (8)	C11—C12	1.383 (8)
C3—C6	1.509 (8)	C12—C13	1.386 (8)
C3—C5	1.513 (8)	C12—H12	0.9300
C5—H5A	0.9600	C13—H13	0.9300
			
C2—O1—C3	118.4 (5)	C3—C6—H6B	109.5
C4—O2—C3	118.9 (5)	H6A—C6—H6B	109.5
C7—N1—C8	125.2 (6)	C3—C6—H6C	109.5
C7—N1—H1N	117 (5)	H6A—C6—H6C	109.5
C8—N1—H1N	118 (5)	H6B—C6—H6C	109.5
C7—C1—C2	122.1 (6)	N1—C7—C1	126.0 (6)
C7—C1—C4	116.9 (6)	N1—C7—H7	117.0
C2—C1—C4	120.8 (6)	C1—C7—H7	117.0
O4—C2—O1	118.0 (6)	C13—C8—C9	119.7 (6)
O4—C2—C1	125.5 (6)	C13—C8—N1	117.7 (5)
O1—C2—C1	116.4 (5)	C9—C8—N1	122.6 (6)
O1—C3—O2	111.2 (5)	C10—C9—C8	120.5 (6)
O1—C3—C6	110.4 (5)	C10—C9—H9	119.7
O2—C3—C6	109.8 (5)	C8—C9—H9	119.7
O1—C3—C5	105.7 (5)	C9—C10—C11	119.0 (6)
O2—C3—C5	106.1 (5)	C9—C10—H10	120.5
C6—C3—C5	113.6 (6)	C11—C10—H10	120.5
O3—C4—O2	117.9 (6)	C12—C11—C10	121.3 (6)
O3—C4—C1	125.5 (6)	C12—C11—Br1	119.5 (5)
O2—C4—C1	116.4 (6)	C10—C11—Br1	119.2 (5)
C3—C5—H5A	109.5	C11—C12—C13	119.5 (6)
C3—C5—H5B	109.5	C11—C12—H12	120.3
H5A—C5—H5B	109.5	C13—C12—H12	120.3
C3—C5—H5C	109.5	C8—C13—C12	120.0 (6)
H5A—C5—H5C	109.5	C8—C13—H13	120.0
H5B—C5—H5C	109.5	C12—C13—H13	120.0
C3—C6—H6A	109.5		
			
C3—O1—C2—O4	162.6 (5)	C2—C1—C4—O2	10.3 (8)
C3—O1—C2—C1	−20.6 (8)	C8—N1—C7—C1	−179.4 (6)
C7—C1—C2—O4	−6.4 (10)	C2—C1—C7—N1	2.0 (10)
C4—C1—C2—O4	167.6 (6)	C4—C1—C7—N1	−172.3 (6)
C7—C1—C2—O1	177.0 (5)	C7—N1—C8—C13	−168.0 (5)
C4—C1—C2—O1	−8.9 (8)	C7—N1—C8—C9	11.5 (9)
C2—O1—C3—O2	46.4 (7)	C13—C8—C9—C10	2.0 (9)
C2—O1—C3—C6	−75.7 (7)	N1—C8—C9—C10	−177.5 (5)
C2—O1—C3—C5	161.1 (5)	C8—C9—C10—C11	0.1 (9)
C4—O2—C3—O1	−44.9 (7)	C9—C10—C11—C12	−2.3 (9)
C4—O2—C3—C6	77.5 (6)	C9—C10—C11—Br1	178.8 (5)
C4—O2—C3—C5	−159.4 (5)	C10—C11—C12—C13	2.3 (9)
C3—O2—C4—O3	−166.3 (5)	Br1—C11—C12—C13	−178.7 (4)
C3—O2—C4—C1	17.6 (7)	C9—C8—C13—C12	−1.9 (8)
C7—C1—C4—O3	8.9 (9)	N1—C8—C13—C12	177.6 (5)
C2—C1—C4—O3	−165.4 (6)	C11—C12—C13—C8	−0.2 (8)
C7—C1—C4—O2	−175.3 (5)		

### Hydrogen-bond geometry (Å, °)

**Table:**

*D*—H···*A*	*D*—H	H···*A*	*D*···*A*	*D*—H···*A*
N1—H1N···O4	0.98 (7)	2.06 (8)	2.770 (7)	128 (6)
C9—H9···O3^i^	0.93	2.49	3.345 (8)	152

Symmetry codes: (i) −*x*+2, −*y*, −*z*+2.
